# A Quality Improvement Approach to Reduce Unplanned Extubation in the NICU While Avoiding Sedation and Restraints

**DOI:** 10.1097/pq9.0000000000000346

**Published:** 2020-09-25

**Authors:** C. Briana Bertoni, Thomas Bartman, Gregory Ryshen, Brandon Kuehne, Marissa Larouere, Leslie Thomas, Erin Wishloff, Edward Shepherd, Julie Dillard, Leeann R. Pavlek, Mohannad Moallem

**Affiliations:** From the * Division of Outreach Medicine, Children’s National Medical Center, Washington, D.C.; † Department of Pediatrics, Reston Hospital Center, Reston, V.I.; ‡ Department of Neonatology, Nationwide Children’s Hospital, Columbus, Ohio; § Neonatal Intensive Care Department, King Abdulaziz Medical City, Riyadh, Saudi Arabia.

## Abstract

The unplanned extubation (UE), a common adverse event in the neonatal intensive care unit (NICU), may result in airway trauma, cardiopulmonary resuscitation, and, in extreme cases, death. As part of the Nationwide Children’s Hospital NICU’s effort to optimize NICU graduates’ neurodevelopmental outcomes, skin-to-skin care of intubated infants is encouraged, while sedation and restraints to prevent UE are strongly discouraged. This project aimed to decrease the UE rate from 1.85 to 1.5 per 100 endotracheal tube (ETT) days.

**Methods:**

The project occurred in a 114-bed, level-IV NICU with approximately 850 admissions per year and 100% outborn infants. A multidisciplinary team began biweekly meetings to review all UE events, later separating these into preventable and nonpreventable. Important ongoing tests of change included assigning a single process owner for UE reporting, ensuring proper ETT securement, and using 2 clinical staff during patient and/or ETT manipulation.

**Results:**

Early in the project, enhanced detection led to an increased rate from 1.85 to 3.26 per 100 ETT days. However, identifying preventable events empowered staff to decrease the frequency to 2.03 per 100 ETT days. In August 2017, an ETT taping method change produced an increase in special causes due to decreased compliance. However, when securement methods were enhanced, noncompliance reversed and is now trending favorably.

**Conclusions:**

Decreasing UE in a neurodevelopmentally friendly unit, which avoids sedation and restraints, is challenging. Using a multidisciplinary quality improvement approach and after appropriately capturing events, we reduced UE, with the highest impact of intervention being ETT securement standardization.

## Abstract 2

Supplemental Digital Content is available in the text.

## INTRODUCTION

The neonatal intensive care unit (NICU) at Nationwide Children’s Hospital includes 114 beds caring for a heterogeneous population, including premature neonates as young as 22 weeks’ gestation, newborns with congenital (including airway) malformations, and patients up to 2 years old with bronchopulmonary dysplasia. Many NICU patients require prolonged invasive mechanical ventilation, during which unintentional endotracheal tube (ETT) dislodgment poses a risk. As part of our institution-wide goals to eliminate preventable harm, the Zero Hero initiative,^1,2^ as well as part of Solutions for Patient Safety (SPS),^3^ we sought to reduce the unplanned extubation (UE) rate in our NICU.

UEs lead to short- and long-term complications. In a large level-IV NICU,^4^ Hatch et al^5^ demonstrated that emergent intubations (most commonly occurring after UE) have a 4-fold odds of increase in adverse events when compared with elective and urgent intubations. Cardiovascular collapse occurs in 20% of UEs for infants and children admitted to the NICU, pediatric intensive care unit, or cardiac intensive care unit, with 83% of these cases requiring cardiopulmonary resuscitation.^6^ Complications from repeated intubation attempts performed emergently include intraventricular hemorrhage, tracheal injury, and pulmonary injury.^7^ Additionally, Thomas et al^8^ demonstrated that repeated intubations after UE is a potential risk factor for severe acquired subglottic stenosis in NICU graduates.

Similar institutions have reduced their UE rate following quality improvement (QI) initiatives focused on enhanced UE recognition, staff education, and standardizing practices. Merkel et al^9^ reported a significant improvement in UE from 2.38 to 0.41 per 100 ETT days following multiple Plan, Do, Study, Act (PDSA) cycles, with the most significant reduction in UE rate seen after implementing the use of mittens on the hands of infants older than 34 weeks’ postmenstrual age and a visible progress display. Similarly, Crezeé et al^10^ demonstrated a decrease in UE from 1.15 to 0.54 per 100 ventilator days following similar interventions, but including the use of procedural sedation to resecure ETTs, in a level-IV NICU with a low baseline UE rate. Our project, however, differs significantly from those listed above by avoiding sedation and restraints in intubated patients and the active encouragement of skin-to-skin care for all infants.

As part of the Nationwide Children’s Neonatal Network’s effort to ensure optimal neurodevelopmental outcomes, skin-to-skin care of intubated infants (including those on high-frequency ventilation) is encouraged, while sedation and restraints to prevent UE are strongly discouraged. Our older patients are expected to perform developmentally appropriate tasks with the arms and hands, including grabbing objects. While the multidisciplinary team was charged with making interventions to reduce UE and the associated risks, we decided interventions impairing long-term neurodevelopmental outcomes would be unacceptable.

This QI project aimed to decrease the UE rate in infants admitted to the Nationwide Children’s Main Campus NICU from 1.85 to 1.5 per 100 ETT days by December 31, 2017, and sustain this decrease for 12 months.^11^

## METHODS

### Context

This project occurred at Nationwide Children’s Hospital Main Campus NICU, a 114-bed, level-IV NICU with approximately 850 all outborn admissions per year. Like other level-IV NICUs, Nationwide Children’s Hospital has many neonates requiring prolonged intubation secondary to various diagnoses, including extreme prematurity and congenital anomalies. Nationwide Children’s Hospital’s NICU also has 24 beds dedicated to caring for patients with severe bronchopulmonary dysplasia.^12,13^ Nationwide Children’s Hospital participates in SPS, a national collaborative of more than 130 children’s hospitals working to eliminate preventable harm. This project occurred as part of the institution’s Zero Hero^1,2^ effort to eliminate preventable harm and an SPS^3^ initiative to decrease UE.

### Interventions

In January 2014, a monthly staff meeting was initiated across the hospital’s intensive care units to discuss individual UE events and possible causation factors. The teams in the hospital used the Institute for Healthcare Improvement methodology implementing multiple PDSA cycles.^14^

**Fig. 1.**
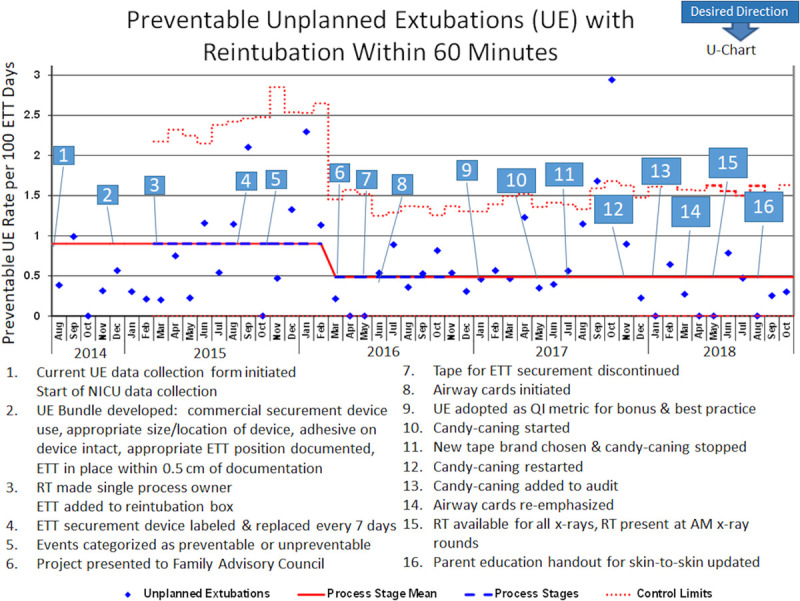
U-Chart for preventable unplanned extubations requiring reintubation within 60 minutes.

In May 2014, a multidisciplinary NICU-specific team, including a QI specialist, neonatal nurse practitioners, nurses, a nurse educator, respiratory therapists (RTs), and physicians, began meeting biweekly to review each UE event and associated failure modes. Our working group devised multiple interventions (Table 1) and UE event review identified high-risk elements, which became the UE prevention bundle in December 2014. Audits were performed to track specific interventions, including bundled elements and taping technique compliance. The team, however, struggled with event capture. In February 2015, a questionnaire was placed in all intubation boxes to improve reporting, evaluate potential factors that contributed to each event, and identify immediate adverse outcomes, such as the need for cardiopulmonary resuscitation (**See figure 1, Supplemental Digital Content**, which displays unplanned extubation questionnaire, **http://links.lww.com/PQ9/A208**). At this time, RTs began owning the reporting process.

**Table 1.** Intervention Timeline

Date	Intervention(s)
January 2014	• Began participation in Solutions for Patient Safety UE Initiative
May 2014	• Biweekly NICU multidisciplinary team meetings began
August 2014	• Start of NICU data collection
	• Current UE questionnaire form initiated, including binary responses related to best practices for ETT position maintenance (see figure 1, Supplemental Digital Content)
December 2014	• “UE Bundle” developed
	**○** Commercial securement device use
	**○** Appropriate size and location of the device
	**○** Device adhesive intact
	**○** Appropriate position of ETT documented
	**○** ETT in place within 0.5 cm of documentation
February 2015	• Respiratory therapist made responsible for event capture
	• ETT forms added to intubation boxes
July 2015	• Labeling securement device and replacement every 7 days added to bundle based on manufacturer’s recommendation
October 2015	• Categorization of events as preventable (caregiver engaged with infant or manipulating ETT tube at the time of event) or unpreventable to increase staff empowerment
March 2016	• Project presented to Family Advisory Council for parental feedback
May 2016	• Tape for ETT securement to device discontinued by manufacturer
May 2016 to June 2017	• Multiple tape brands tried
June 2016	• “Airway Cards” initiated at bedsides of ventilated patients (including ETT size and depth, cuffed versus uncuffed): Trouble with adaptation and implementation
January 2017	• UE adopted as NICU service line QI metric for bonus and best practice
May 2017	• Candy-caning started as standard of care
July 2017	• New tape chosen as replacement and candy-caning taping technique stopped
November 2017	• Candy-caning reinitiated due to increased events related to tube sliding through tape
January 2018	• Candy-caning added to audit
	• Encouraged “airway guardian” use—ETT sole responsibility
March 2018	• Airway cards re-emphasized
	• Respiratory care assistants became responsible to place and fill out airway cards
April 2018	• Radiology collaborative—initiated “best position during x-ray” to standardize patient positioning and ETT placement during films
May 2018	• RT presence at x-rays (confirm placement and monitor ETT)
	• RT presence at morning x-ray rounds to discuss ETT placement on film
July 2018	• Airway cards transitioned to RT responsibility and updated to include the following:
	**○** ETT size and depth
	**○** Due date for ETT securement device change
	**○** ETT depth and patient weight at last x-ray
	**○** Date, time, and RT initials from last airway card update
August 2018	• Parent education handout for skin-to-skin updated with information about intubated infants

In July 2015, to decrease the UE preventable events attributed to loose securement devices, the NICU implemented manufacturer guidelines and added replacing the ETT securement device every 7 days to the prevention bundle. Before this intervention, the time between securement device changes was not standardized.

To achieve the aim of the project without compromising Nationwide Children’s Hospital’s Neonatal Network neurodevelopmental goals, we tried multiple options to address ETT securement, while allowing infants to be awake and interactive (Table 1). Noteworthy interventions included defining preventable versus nonpreventable UE and “candy-caning” the tape used to secure the ETT to the commercial device. We defined preventable UE as any UE during which a caregiver (parent or staff) was engaged with the infant or was manipulating the tube. We defined unpreventable UE as those caused by normal infant movements that cannot be prevented without restraint or sedative medications. Candy-caning is a method of wrapping the tape around the platform of the securement device and around the ETT in an upward fashion to increase the contact surface area of the adhesion with the ETT device and to prevent tube migration through dilated or moist tape. The application of the candy-caning method began in May 2017 and stopped in July 2017 with the introduction of a new tape thought to have sufficient adhesive properties, which would not require this technique. The candy-caning technique was reintroduced in November 2017 with auditing beginning in January 2018, when UE events increased without it.

### Measures

The team collected multiple measures throughout the project. Outcome measures were the overall UE rate (the number of UEs per 100 ETT days) and the preventable UE rate (the number of preventable UEs per 100 ETT days). The SPS defines UE as any unintentional ETT dislodgement from the trachea. This definition includes any patient with an ETT but excludes those with tracheostomy tubes and events occurring outside the hospital during transport.^3^ We determined the total number of ETT days by adding up the number of days all infants in the unit were intubated. The process measures included UE bundle compliance (completion of all bundled elements) and compliance with the candy-caning technique while taping. The bundled procedures were updated throughout the project, as noted in Table 1. Current bundled elements include commercial securement device use, appropriate size and location of securement device, device labeling with the date of placement, securement device change every 7 days, intact device adhesive, and appropriate ETT position (within 0.5 cm) per documentation. The balancing measure was the reintubation rate after UE.

### Analysis and Ethical Considerations

Microsoft Excel 2010 was used for data analysis and display. Control charts were developed using a Microsoft Excel plug-in coded by our statistician. We used the American Society for Quality special-cause rules to examine the control charts and separate common-cause versus special-cause variation.^15^ The institutional review board waived this project as QI work.

## RESULTS

Baseline data from August 2014 to February 2015 showed 1.85 total UE events per 100 ETT days. In March 2015, we designated a single process owner for UE event capture and began placing the UE huddle forms in the reintubation boxes. We noted special-cause variation and had an increase to 3.26 total UE events per 100 ETT days, most likely due to improved event capture from these interventions. In October 2015, we began to distinguish preventable from nonpreventable events. A retrospective review of reported events distinguished preventable versus unpreventable events through the baseline period. The preventable UE (Fig. 1) trend mirrored the total UE trend.

**Fig. 2.**
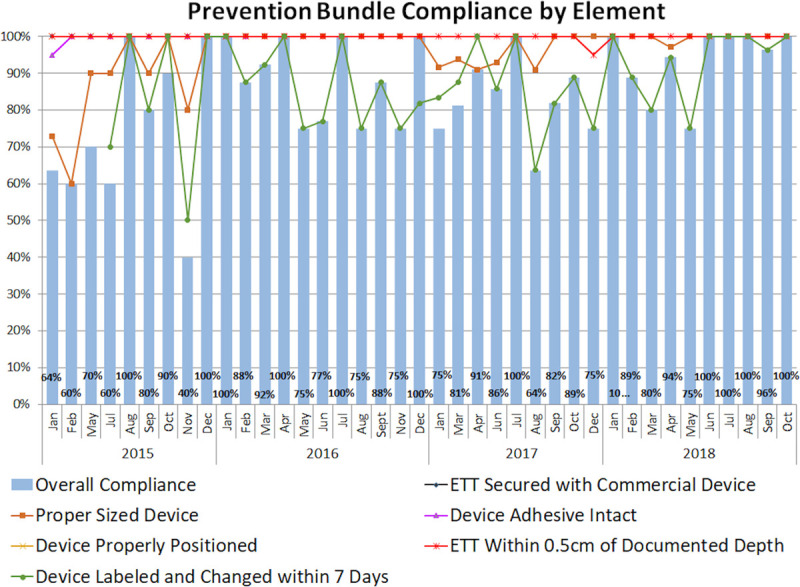
Compliance with unplanned extubation prevention bundle overall and by element.

The prevention bundle was audited every 2–4 weeks (Fig. 2). Review of UE huddle forms revealed that 20% of events were attributed to an equipment issue, including loose tape and loose or expired securement devices. In spite of adding securement device change every 7 days to the prevention bundle in July 2015, compliance with securement device change every 7 days did not improve until March 2016 (85.9% before versus 87.9% after, *P* = 0.048). Furthermore, in March 2016, the project was presented to the Family Advisory Council for parental feedback and buy-in. The combination of increased compliance with securement device change every 7 days and increased parental involvement decreased overall and preventable UE rates. Since March 2016, the total UE rate has remained 2.03 events per 100 ETT days.

**Fig. 3.**
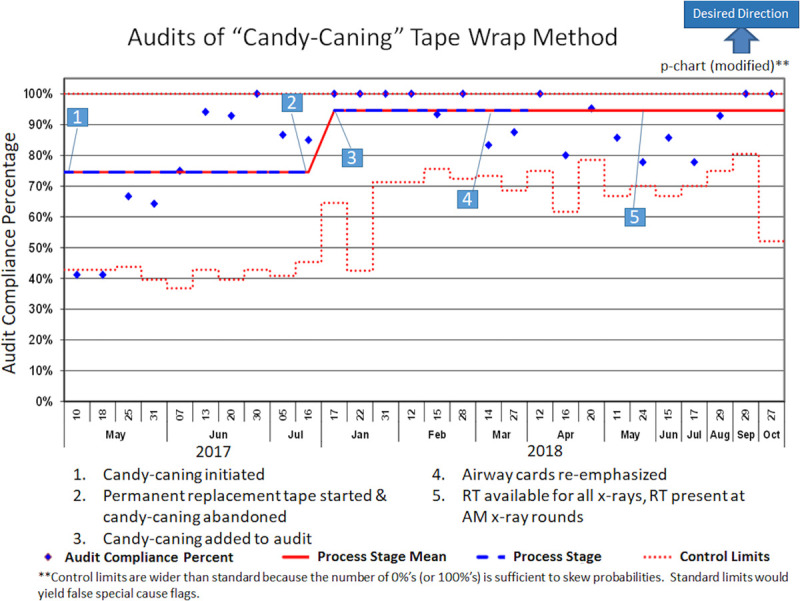
Compliance audits with the candy-caning methodology for taping the endotracheal tube to the securement device. Candy-caning is a method of wrapping the tape up the ETT to increase the contact surface area of the adhesion with the ETT device and to prevent tube migration through dilated or moist tape.

In late summer 2016, after the vendor discontinued the ETT tape, biweekly event reviews noted an increase in UE events related to the ETT becoming detached from the securement device. As a result, an intervention to enhance the contact surface area of the adhesion of the ETT to the securement device (“candy-caning”) was developed, as discussed in the section Interventions. Abandoning the candy-caning technique following the permanent introduction of a new tape brand (Fig. 1, No. 11) resulted in a special-cause variance with high UE rates in September and October 2017. Therefore, candy-caning was reinstituted in November 2017, and auditing, including random independent evaluation for appropriate technique, started in January 2018 (Fig. 3, No. 3). Though not a special cause, there were zero preventable events in 3 months in early 2018. There were no identified explanations for the slightly increased rates during June and July 2018, and these numbers remained within the expected common-cause variation. During this period, 16.3% of events occurred during parental holding or during skin-to-skin care, so the parent education handout about skin-to-skin care was updated in August 2018 to include care of intubated patients (Fig. 1, No. 16).

## DISCUSSION

This QI project aimed to reduce UEs in a busy, level-IV, all referral center as part of the institutional road map eliminating adverse events, “Zero Hero.”^1,2^ The challenge was to achieve this aim in a neurodevelopmentally friendly unit, which encourages skin-to-skin care in all infants and avoids practices that compromise NICU graduates’ neurodevelopmental outcomes, such as routine sedation and restraint use in intubated babies. We achieved our goal of reducing all UE by reducing “preventable UE” through a multidisciplinary team effort to study and adapt multiple QI interventions.

At first, the UE rate increased noticeably early in the project in 2015. The increase was likely due to improved detection, which we achieved after developing a detailed questionnaire for each UE (**see figure 1, Supplemental Digital Content**, which displays unplanned extubation questionnaire, **http://links.lww.com/PQ9/A208**) and assigning the patient’s respiratory therapist as a “single owner” for event capture. Similar findings of increased rates after implementing new detection techniques have been shown previously in UE research.^16^ One study has documented underreporting and underdetection of adverse events, in general, in the NICU, including UEs.^17^ Studies have also showed marked institutional variability in defining and reporting UEs, making it difficult to compare rates between hospitals directly.^18,19^ In this study, we report the rate of preventable UE that required reintubation within 60 minutes, leading to relatively lower rates of UE when compared with studies reporting only total UE rates rather than focusing on a specific subgroup of events.

Biweekly multidisciplinary event review identified ETT securement and taping as the root cause of UE in many cases, which led to adopting and standardizing ETT taping, using a “candy-caning” taping technique to the securement device. With improved candy-caning compliance, a significant reduction in the preventable UE rate occurred, making candy-caning one of our most effective interventions. Notably, around the time that candy-caning began, differentiation of preventable versus unpreventable events also likely led to enhanced staff empowerment and increased buy-in, as discussed below.

Although multiple previously published studies demonstrated the ability to reduce UE in the NICU using QI methods,^9,10^ our QI project differs from these projects by avoiding tools that may affect an infants’ short- or long-term neurodevelopmental outcomes. We avoided sedative and paralytic drug use, anything that might restrain the infants’ normal head, hand, or body movement, and anything that may affect their normal sensory development, such as mittens. Besides, mittens are considered a restraint when used in any patient older than 28 days at our institution. The majority of patients who experienced a UE during this period were intubated beyond the first 28 days of life.

Furthermore, our team is the first to distinguish UEs as preventable and unpreventable in response to staff feedback about the project. We acknowledge that unrestrained and intubated NICU patients will experience UEs because of developmentally appropriate hand-to-mouth movements and the normal grasping reflex. Although we counted these events in our total UE rate and tried to decrease their incidence, we consider such events unpreventable. We believe that neurodevelopmentally unfriendly measures might be required to prevent them, and we were not willing to accept this trade-off. Dividing events led us to focus on eliminating the preventable UE, a task that staff deemed inspiring, which led to a decrease in the total UE rate.

Despite the demonstrated outcome improvements, this project had limitations. Before project initiation, a mechanical ventilation weaning protocol to expedite extubation for extremely low-weight infants was developed, which our UE committee emphasized during this project. This protocol could have decreased the overall UE number by reducing opportunities; however, mechanical ventilation weaning should not affect the UE rate, as this ratio factors in the total number of ETT days. Another limitation is that, although we emphasize the avoidance of using sedative medications to prevent UE and this is the general practice in our NICU, we do not have data on the use of sedation in this population.

This project’s generalizability also has potential limitations. First, we understand that, depending on staffing and job responsibilities, integrating RT into UE tracking and evaluation may be difficult for all units. Our team feels strongly that involving frontline staff with the most access to and experience with the ETT, namely RT and bedside nursing, is the best way to keep people engaged and excited about the project. We also recognize that some units have lower UE rates due to sedation or restraints for prevention and may hesitate to abandon these practices. However, we feel that the neonatal staff’s goal is not only to optimize outcomes during the NICU stay but to enhance both short-term and long-term results for infants requiring intensive care unit admission after birth. We demonstrate the feasibility of reducing UEs while still providing neurodevelopmentally friendly care by avoiding restraints and sedation and encouraging skin-to-skin care for all intubated neonates.

In spite of improvement shown with this project, we desire additional improvement. Given that almost 20% of total UE events occurred during holding or skin-to-skin care, including transferring the baby back to the crib, small PDSA cycles geared toward decreasing UEs during skin-to-skin care, including increased parental involvement and education, are ongoing. Furthermore, PDSA cycles to optimize accurate ETT depth and to minimize unnecessary ETT manipulation are being developed in close collaboration with our Radiology colleagues, based on analysis of x-ray technique and ETT position during clinically indicated films.

## CONCLUSIONS

This QI project presents an effort to tackle a significant health care event, UEs, using neurodevelopmental friendly tools. It enriches the QI literature with a new logical definition of preventable UE, which might need more focus to improve the overall UE rate.

## DISCLOSURE

The authors have no financial interest to declare in relation to the content of this article.

## Supplementary Material


