# Supplementary material for: A Quality Improvement Approach to Reduce Unplanned Extubation in the NICU While Avoiding Sedation and Restraints
Source: Pediatr Qual Saf. 2020 Sep 25;5(5):e346. doi: 10.1097/pq9.0000000000000346 (PMC8487773; doi:10.1097/pq9.0000000000000346)
Supplement: Supplementary file 1 [file pqs-5-e346-s001.pdf]

UNPLANNED EXTUBATION QUESTIONNAIRE

RCP completing form:	Place Patient Sticker Here
Date of unplanned extubation:	
Time of unplanned extubation:	
Date / Time of huddle:	
RN # of patients in assignment:	
RCP# of patients in assignment	
RCP # of ventilatory units assigned (cpap/vent):	

****Brief Description of the Event:			
Individuals present for huddle process:			
Securing device (circle one)	NeoBar		Tape
Ventilator type (circle one)	Conventional		HFOV
	YES	NO	NA
1. Was RN present during the extubation?			
2. Was RCP present during the extubation?			
3. Was the patient reintubated?			
4. Was NeoBar correctly applied with adequate adhesive integrity?			
5. ETT stabilization: two licensed caregivers present for retape/repositioning?			
6. Copious secretions present?			
7. Possible/Confirmed ETT plugging?			
8. Did unplanned extubation occur during suctioning?			
9. Was patient swaddled?			
10. Was patient being held?			
11. Did event occur during a procedure or transfer?			
12. Were two licensed caregiver transfer/care utilized?			
13. Was the ventilator circuit secured? Twill? Angelframe?			
15. Was extubation confirmed by a Pedi Cap?			
16. Was NeoBar dated with a born-on date?			
17. Is date on NeoBar within past 7 days?			
18. Was a physical examination done to confirm extubation?			
19. Although unplanned, was this an elective extubation (tube purposely pulled by team due to deteriorating patient condition)?			

Please comment on the circumstances surrounding the extubation and elaborate on any box highlighted above:

De-Saturation?: Yes No, ______Minutes (if Yes)

Bradycardia?: Yes No, ______Minutes (if yes)

CPR Given?: *Yes No, ______Minutes (if yes)

****If yes, remind to team to fill out unit based code form***
